# Ectopic Intrathyroidal Parathyroid Adenoma Presenting With Osteoporotic Fractures in a Young Man: A Case Report

**DOI:** 10.7759/cureus.47461

**Published:** 2023-10-22

**Authors:** Inês Cosme, Ema Nobre, André Travessa, Catarina Santos, José Rocha, Dolores Presa, Ana P Barbosa

**Affiliations:** 1 Endocrinology, Diabetes and Metabolism, Centro Hospitalar Universitário Lisboa Norte, Lisboa, PRT; 2 Genetics, Centro Hospitalar Universitário Lisboa Norte, Lisboa, PRT; 3 Surgery, Centro Hospitalar Universitário Lisboa Norte, Lisboa, PRT; 4 Pathology, Centro Hospitalar Universitário Lisboa Norte, Lisboa, PRT

**Keywords:** primary hyperparathyroidism (phpt), men osteoporosis, intrathyroidal parathyroid adenoma, fragility fractures, ectopic parathyroid adenoma

## Abstract

Primary hyperparathyroidism (PHPT) can be associated with osteoporosis (OP) and fractures. We present a case of a 49-year-old male referred to our osteoporosis outpatient clinic due to a right femur osteoporotic fracture. At the age of 38, a right plantar nodular lesion was excised, and its histology was compatible with a deep dermis nodule formed by mononuclear and giant osteoclast-like cells. He has reported osteoporotic fractures since age 39 and renal colic episodes since age 45. His father had lipomas and renal colic episodes, and his paternal grandmother had lipomas. The laboratory evaluation was compatible with PHPT. A cervical ultrasound showed a 10mm single solid nodule in the left thyroid lobe, strongly hypoechogenic, with microcalcifications. Its cytology showed parathyroid tissue without atypia. Parathyroid scintigraphy had no uptake. A dual-energy X-ray absorptiometry scan showed a femoral neck Z-score of -4.3. He started alendronate/cholecalciferol (70mg/5600IU) weekly. He was submitted to a left hemithyroidectomy. Its histology showed an intrathyroidal parathyroid adenoma. Ectopic parathyroid adenomas are rare, of which 0.7%-6% are intrathyroidal. The excised foot lesion could be a brown tumour. Furthermore, calcium metabolism evaluation at that time might have allowed a PHPT diagnosis and its morbidity prevention. Osteoporotic fractures in young men must alert to secondary OP.

## Introduction

Secondary osteoporosis (OP) is defined as bone loss with microarchitectural alterations leading to fragility fractures caused by medication or underlying diseases. Medical disorders responsible for secondary OP include endocrine diseases, gastrointestinal/biliary tract disorders, renal diseases, cancer and prolonged immobilisation [1]. Endocrine disorders are the most frequent cause of secondary OP [2]. It is recommended to exclude secondary OP in cases of recent fractures, multiple fractures or very low bone mass density in women before menopause and men under 70 years old [3].

In assessing a man with suspected OP, it is recommended to consider secondary causes because 50%-70% of male OP cases have a secondary aetiology [4]. Concerning the aetiology of male secondary OP, bariatric surgery, hypercalciuria, hyperparathyroidism, inflammatory bowel disease and primary hypogonadism are possible causes. Glucocorticoids are the most common iatrogenic cause, and androgen deprivation therapy for prostate cancer is another relevant iatrogenic cause [5].

Primary hyperparathyroidism (PHPT) is diagnosed, in most cases, as an incidental finding on routine laboratory tests [6], and the majority of PHPT patients are asymptomatic [7]. Nevertheless, some non-specific symptoms can be presented at the diagnosis, such as fatigue, joint aches, weakness, mild depression and difficulty in concentrating [6]. As stated in a 15-year prospective follow-up study, one-third of the PHPT patients, without treatment, will have overt features such as osteopenia/OP, kidney stones and worsening hypercalcaemia [6].

Hyperparathyroidism leads to bone loss and increased fracture risk, particularly in the cortical bone. Studies describe a prevalence of OP due to PHPT ranging from 39% to 62.9%, with the peripheral skeleton preferentially affected [8]. Osteoporotic fractures can be caused by persistent or long-term severe hyperparathyroidism [2]. The fracture prevalence is heterogeneous between studies. However, it is stated that approximately 3% of the newly diagnosed pathological fractures are related to hyperparathyroidism [9]. We present a case of a young male with a fragility femoral neck fracture and PHPT. This article was previously presented as a meeting abstract at the 73ª Reunião Anual da SPEDM, Congresso Português de Endocrinologia in February 2023.

## Case presentation

A 49-year-old man was referred to our osteoporosis outpatient clinic after a right femoral neck fragility fracture (after he had a fall from his height). At the age of 38, this patient had an excision of a nodule in his right plantar foot that was incomplete in length and depth performed at the dermatology clinic. Its histological report showed ‘a nodule constituted by mononucleated cells without atypia and giant cells like osteoclast in the deep dermis, compatible with tenosynovial giant cells tumour’. After that procedure, he quit the medical follow-up.

**Figure 1:**
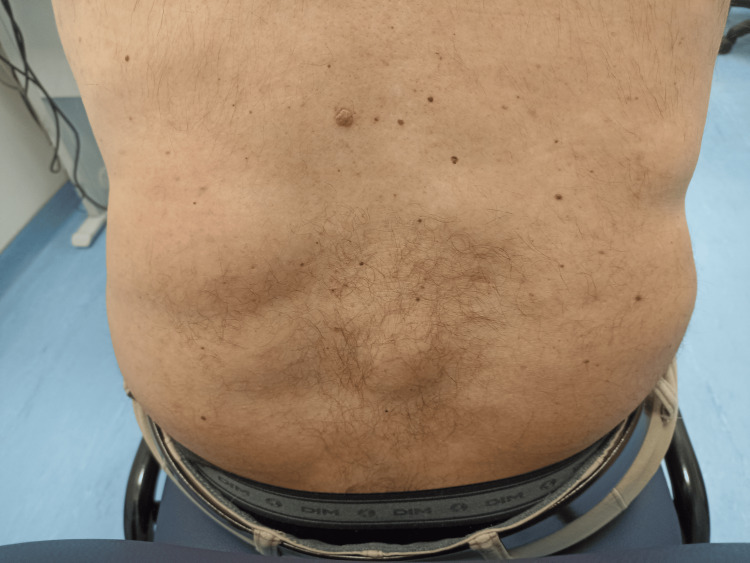
Lipomas in patient's back

He also reported progressive complaints of several renal colic episodes associated with generalised muscle weakness, fatigue, diffuse bone pain and multiple non-associated trauma fractures of the right shoulder and fingers of both hands since the age of 39. He denied other diseases and chronic medication. His father had some renal colic episodes and several chest lipomas, and his paternal grandmother also had a few chest lipomas. Due to the painful complaints, he reported a marked limitation of his daily activities and quality of life. His physical examination was unremarkable, except for several scattered lipomas (Figure 1).

Initial blood tests were compatible with PHPT. The pituitary function and neuroendocrine hormones were normal (Table 1).

**Table 1:** Initial laboratory evaluation PTH: parathyroid hormone, 25(OH)D: 25 hydroxyvitamin D, HbA1C: haemoglobin A1C, TSH: thyroid-stimulating hormone, FT4: free thyroxine, FSH: follicle-stimulating hormone, LH: luteinizing hormone, ACTH: adrenocorticotropic hormone, IGF-1: insulin-like growth factor 1.

Analyte	Result	Normal range
PTH	209 pg/mL	14-72
Calcium	10.7 mg/dL	8.6-10.2
Phosphate	1.4 mg/dL	2.5-4.5
Calcium in 24h urine	174 mg/24h	100-300
Phosphate in 24h urine	739 mg/24h	400-1,300
25(OH)D	21.4 ng/mL	>30
Bone alkaline phosphatase	88.2 µg/L	5.5-20.1
Creatinine	0.91 mg/dL	0.7-1.2
Estimated glomerular filtration rate	98 ml/min/1.73m^2^	>60
Fasting glycaemia	111 mg/dL	<126
HbA1C	5.8%	<6.5%
Insulin	20 µU/mL	2.6-25
TSH	1.23 µU/mL	0.3-4.2
FT4	1.43 ng/dL	0.85-1.7
FSH	12.6 U/L	1.5-12.9
LH	9.01 U/L	1.3-9.8
Total testosterone	499 ng/dL	190-740
Prolactin	18.6 ng/mL	4-15
ACTH	20.4 pg/mL	9-52
Cortisol	14.8 µg/dL	10-20
IGF-1	186 ng/mL	80.6-200
Calcitonin	7.28 pg/mL	<14.5
Urinary metanephrines	Metanephrine: 233 µg/24h	<320
	Normetanephrine: 685 µg/24h	<865
	3-methoxytyramine: 172.4 µg/24h	<460
Chromogranin A	93.3 ng/mL	<102
Gastrin-17	1.5 pmol/L	1.7-7.6

**Figure 2:**
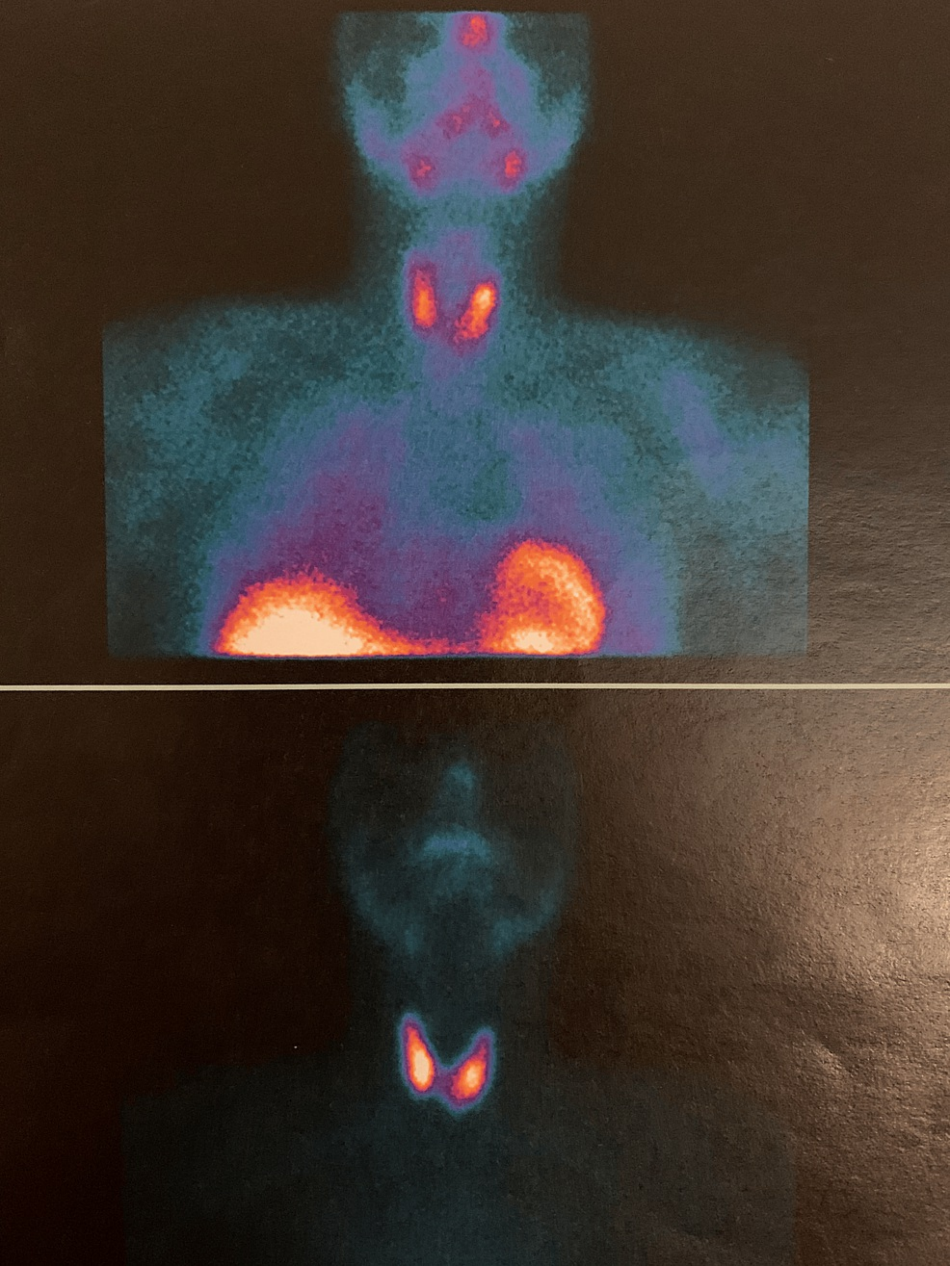
Parathyroid scintigraphy

The cervical ultrasonography documented a unique 10mm solid and markedly hypoechogenic nodule with microcalcifications in the left thyroid lobe. Its fine-needle aspiration cytology (FNAC) showed parathyroid tissue without atypia. Its immunochemistry was positive for parathyroid hormone (PTH), GATA3 and AE1/AE3 and negative for TTF1 and thyroglobulin. The PTH measurement in the FNAC washout was >5,000 pg/mL. The sestamibi parathyroid scintigraphy was negative (Figure 2).

A dual-energy X-ray absorptiometry scan revealed a mean Z-score of -4.3 in the femoral neck and the total hip and a mean Z-score of -3.1 in the lumbar spine (L1-L4). There were no vertebral fractures on his dorsal-lumbar X-ray. Bone scintigraphy demonstrated microfractures in several costal arches. The abdominal computerised tomography scan showed left kidney obstructive uropathy and lithiasis. A genetic analysis including next-generation sequencing (NGS) 17 genes panel identified no pathologic form of genetic PHPT. He started alendronate/cholecalciferol (70 mg/5,600 IU) weekly and was submitted to a left hemithyroidectomy. Histology demonstrated an intrathyroidal parathyroid adenoma without other alterations in the remaining parenchyma.

## Discussion

This case illustrates a probable long progressive history of PHPT ending with a femoral neck fracture. Despite PHPT is diagnosed by an abnormal result in biochemical screening or by non-specific symptoms in most cases, there are rare cases of advanced PHPT that are presented with bone disease, such as osteitis fibrosa cystica, osteomalacia and, rarely, brown tumours [10].

In this report, we described a case of a young man with a long history of fragility fractures. We consider that this progressive history in a young man must signal secondary OP [11]. The crude fracture rate in patients with PHPT has been documented to be 15/1,000 person-year compared to 8/1,000 in controls [12]. Fractures in PHPT patients are due to brown tumours, a rare complication of PHPT. These are benign tumours caused by an imbalance between osteoclastic activity and osteoblastic/fibroblastic proliferation, causing bone resorption, fibrous replacement of the marrow and bone cortex thinning. Brown tumours may be solitary or multiple and develop more frequently in the facial bones and jaws, sternum, pelvis, femur, extremities or clavicles [13].

As found in the patient’s foot, osteoclast-like giant cell lesions can be presented in different types of bone or soft tissue tumours, like brown tumours, mesenchymal phosphaturic or tenosynovial tumours. Despite being similar in histology, its clinicopathological characteristics, prevalence and behaviour differ according to the tumour type [14]. Due to the difficulty in microscopic differential diagnosis, the clinical presentation and the biochemical findings are essential; for instance, an increased serum PTH is essential to establish PHPT diagnosis and, consequently, brown tumour diagnosis [15]. The authors consider that, at the time of the foot excision, it would have been essential to evaluate the calcium metabolism to exclude PHPT and prevent its long-term morbidities. Although the foot is not a usual location for brown tumours, we consider that as the excision was incomplete, the histopathological analysis could be compromised. The biochemical evaluation might be important in supporting the definitive diagnosis.

In a patient with OP due to PHPT, the management of the OP includes not only its medical treatment but also a parathyroidectomy. This surgery can usually reverse the bone loss after one to two years [16]. In this case, we have identified an ectopic parathyroid adenoma with an intrathyroidal location. In about 6%-16% of cases, one or more parathyroid adenomas are in an ectopic position [17]. Intrathyroidal parathyroid adenoma is one of the possible ectopic variants, with an incidence of 0.7% to 6% between the ectopic parathyroid variants [18].

The family’s history of recurrent renal colic and the patient’s younger age may point out a genetic cause of PHPT. Lipomas, a possible cutaneous manifestation of multiple endocrine neoplasia type 1 (MEN1) syndrome [19], made us exclude this genetic aetiology in our patient [20]. An NGS panel analysis of possible genetic causes of PHPT was negative, and pituitary and neuroendocrine hormones evaluation did not reveal any changes. Moreover, the authors intended to evaluate calcium metabolism in the patient’s family; however, it was impossible.

## Conclusions

In conclusion, this case contrasts with the most common presentation of PHPT nowadays as, over the recent decades, its clinical presentation shifted from overt disease to mild biochemical presentation without symptoms. We reported a PHPT presentation causing severe bone disease and OP fractures in a young man. This must alert to secondary OP. It was identified as an ectopic parathyroid adenoma with a rare intrathyroidal location. The biochemical evaluation, at the foot excision time, might have diagnosed PHPT and prevented 10 years of disease morbidity.
